# The Utilisation of the Sac in the Radical Operation for Femoral Hernia1Read at the 35th annual meeting of the Medical Association of Central New York, held at Syracuse, N.Y., October 21, 1902.

**Published:** 1903-01

**Authors:** A. L. Hall

**Affiliations:** Fulton, N. Y.


### The Utilisation of the Sac in the Radical Operation for Femoral Hernia.[1]

By A. L. HALL, M. D., Fulton, N. Y.

FOR a time the opinion prevailed that femoral hernia was less amenable to radical operation than the inguinal variety. Experience has not only demonstrated the incorrectness of this view, but has shown that the per cent. of recoveries is greater in the femoral type.

Of the numerous operations devised for the radical cure of femoral hernia, which have been brought forth from time to time, few have met the general approval of the profession.

In the Bassini and other approved operative methods, occlusion of the canal is effected by suture, after the greater portion of the sac has been excised well up within the canal. As far as I am able to learn, utilisation of the sac for canal closure has never been practised. Several times within the past few years I have operated for strangulated femoral hernia and in each instance a radical cure resulted. In the last two cases a portion

1. Read at the 35th annual meeting of the Medical Association of Central New York, held at Syracuse, N.Y., October 21, 1902.

of the sac was used to occlude the canal. The operative technic employed is briefly described as follows:

The first case was that of a woman aged 72 years in whom acute strangulation had existed for nearly two days. The sac was exposed and opened in the usual way. Intestine, only, was present and being in good condition it was returned within the abdominal cavity. The sac was freed, the dissection being carried for a short distance up the canal, its length reduced to about one-half inch—the approximate depth of the canal, and divided by a longitudinal incision into two equal parts. These flaps, formed from the sac, were deprived of fat, inverted and pushed up the canal until their ends approximated the femoral opening. The lower portion of the invaginated sac was quilted together with fine catgut and the lower canal opening closed by uniting its edges with strong catgut, a considerable portion of the canal being included by the sutures which were continuous. The incision was closed in the usual manner. Recovery was rapid and uneventful. Two years later, the patient died of acute dysentery. There was no return of the hernia.

The second case was an adherent omental hernia of the femoral variety occurring in a woman aged 57 years. The omentum was detached from the sac with some difficulty, found to be healthy and replaced. The technic was the same as in the previous case, the sac being utilised to close the canal. Five years have elapsed since the operation without recurrence.

The limited experience recited proves but little, nevertheless extended trial in the utilisation of the sac for the radical cure of femoral hernia may demonstrate advantages not obtainable by other methods. At least a simplified technic is assured, and there are excellent reasons, I believe, for claiming for it results superior to any obtained by other operative measures.

